# Metabolic interaction between cancer cells and stromal cells according to breast cancer molecular subtype

**DOI:** 10.1186/bcr3472

**Published:** 2013-09-10

**Authors:** Junjeong Choi, Do Hee Kim, Woo Hee Jung, Ja Seung Koo

**Affiliations:** 1 Department of Pathology, Yonsei University Wonju College of Medicine, Ilsan-dong 162, Wonju, Gangwon-do 220-701, South Korea; 2 Department of Pathology, Yonsei University College of Medicine, 50 Yonsei-ro, Seodaemun-gu, Seoul 120-752, South Korea

## Abstract

**Introduction:**

The aim of this study was to investigate the differential expression of markers related to metabolic, mitochondrial and autophagy status in different molecular subtypes of breast cancer.

**Methods:**

Using tissue microarray sections generated from 740 cases of breast cancer, we performed immunohistochemical staining for Glut-1, CAIX, MCT4, ATP synthase, glutaminase, BNIP3, Beclin-1, LC3A, LC3B and p62. Based on the immunohistochemical expression of estrogen receptor (ER), progesterone (PR), HER2, and Ki-67 labeling index, the cases were classified into luminal A, luminal B, HER2 and triple-negative breast cancer (TNBC). We further classified metabolic phenotypes of tumors according to glycolytic status by assessing Glut-1 and CAIX expression as follows: Warburg type: tumor (glycolysis type), stroma (nonglycolysis type); reverse Warburg type: tumor (nonglycolysis type), stroma (glycolysis type); mixed type: tumor (glycolysis type), stroma (glycolysis type); and null type: tumor (nonglycolysis type), stroma (nonglycolysis type).

**Results:**

Expression of Glut-1, MCT4 and LC3A was highest in TNBC and lowest in luminal A type (*P* < 0.001). Tumors were classified into 298 Warburg type (40.3%), 54 reverse Warburg type (7.3%), 62 mixed type (8.4%) and 326 null type (44.0%). The mixed type had a higher histologic grade, ER negativity, PR negativity and Ki-67 index, whereas the null type showed lower histologic grade, ER positivity, PR positivity and Ki-67 index (*P* < 0.001). TNBC constituted the major portion of Warburg and mixed types, and luminal A consisted mainly of reverse Warburg and null types (*P* < 0.001).

**Conclusion:**

Breast cancer is heterogeneous in its metabolic status, and therefore it can be classified into various metabolic phenotypes. Specifically, the Warburg and mixed types had strong associations with TNBC, whereas reverse the Warburg and null types had associations with the luminal type, suggesting a correlation between metabolic phenotype and the biology of breast cancer.

## Introduction

The metabolism of malignant tumors is generally explained by the Warburg effect theory, which describes the metabolic shift from mitochondrial oxidative phosphorylation (OXPHOS) to glycolysis in tumors [1]. Breast cancer is known to be heterogeneous, and the interaction between tumor cells and adjacent stroma is expected to have significant roles in tumor growth and progression. This kind of complex interaction may also exist in the metabolic processes of the tumor. Previous studies suggest a unique metabolic interaction between tumor cells and the stroma of breast cancer, known as the *reverse Warburg effect theory*[2-5]. According to this theory, reactive oxygen species (ROS), such as nitric oxide (NO), generated by tumor cells bring oxidative stress to the stromal cells, leading to mitochondrial dysfunction, autophagy (mitophagy) and increased aerobic glycolysis through hypoxia-inducible factor 1α (HIF-1α) and nuclear factor κB (NF-κB). Lactate generated by stromal cell glycolysis enters tumor cells and promotes tumor cell growth and survival through efficient generation of ATP by OXPHOS in the mitochondria. Cancer-associated fibroblasts (CAFs), stromal cells with loss of caveolin-1 expression, have been implicated in this interaction in breast cancer because loss of caveolin-1 results from increased proteolysis by autophagy [3,5-7]. In addition to the reverse Warburg effect theory, it has been reported that certain types of tumor cells generate ATP through glycolysis as well as OXPHOS, which suggests various features of tumor metabolism [8,9]. Proteins involved in metabolism, mitochondrial function and autophagy may be differentially expressed in both tumor and stromal cells according to the aforementioned theories. These differences are summarized in Table 1.

**Table 1:** **Comparison of metabolism type, mitochondrial status and autophagy status between the Warburg effect theory and reverse Warburg effect theory**^**a**^

	**Warburg effect theory**	**Reverse Warburg effect theory**	
	**Cancer cell**	**Cancer cell**	**Stromal cell**
Metabolism	Glycolysis	OXPHOS	Glycolysis
Mitochondrial status	Dysfunctional	Functional	Dysfunctional
Autophagy status	Not included	Not activated	Activated

^a^OXPHOS, oxidative phosphorylation.

Because breast cancer is heterogeneous with respect to clinical, histopathological and molecular features, several subclassifications have been investigated to stratify tumors with similar characteristics. Gene expression profiles have enabled molecular classification of tumors into luminal A, luminal B, HER2, normal breast-like and basal-like types [10-12]. Studies have also revealed differences in histological and clinical manifestation between different molecular subtypes, such as therapeutic response and prognosis. We hypothesized that the metabolic interaction between tumor cells and stroma may differ according to the molecular subtypes of breast cancer. Because there are limited studies regarding this question, the aim of our present study was designed to investigate the differential expression of markers for metabolic, mitochondrial and autophagy status in different molecular subtypes of breast cancer.

## Methods

### Patient selection

Patients diagnosed with invasive breast cancer treated by surgical resection during the period from January 2002 to December 2006 were included in this study. Patients who received preoperative neoadjuvant chemotherapy or hormonal treatment were excluded. This study was approved by the Institutional Review Board (IRB) of Yonsei University Severance Hospital. The IRB exempted the informed consent from patients. A breast pathologist (JSK) retrospectively reviewed the histology of all cases using hematoxylin and eosin (H&E)-stained slides. The histological grade was assessed using the Nottingham grading system [13]. Clinicopathologic parameters evaluated in each case included patient age at initial diagnosis, lymph node metastasis, tumor recurrence, distant metastasis and patient survival.

### Tissue microarray

On H&E-stained slides of tumors, a representative area was selected and the corresponding spot was marked on the surface of the paraffin block. Using a biopsy needle, the selected area was punched out and a 3-mm tissue core was placed into a 6 × 5 recipient block. Tissue from the invasive tumor was then extracted. More than two tissue cores were extracted to minimize extraction bias. Each tissue core was assigned a unique tissue microarray (TMA) location number that was linked to a database containing other clinicopathologic data.

### Immunohistochemistry

The antibodies used for immunohistochemistry (IHC) in this study are shown in Table 2. Formalin-fixed, paraffin-embedded (FFPE) tissue sections from the TMA were prepared for IHC. Briefly, 5-μm-thick sections were obtained using a microtome, transferred into adhesive slides and dried at 62°C for 30 min. After incubation with primary antibodies, immunodetection was performed with biotinylated anti-mouse immunoglobulin, followed by peroxidase-labeled streptavidin using a labeled streptavidin biotin kit with 3,3′-diaminobenzidine chromogen as the substrate. The primary antibody incubation step was omitted in the negative control. A positive control was included for each experiment: glucose transporter 1 (Glut-1): esophageal carcinoma; carbonic anhydrase IX (CAIX): renal carcinoma; monocarboxylate transporter 4 (MCT4): BCL2/adenovirus E1B 19-kDa interacting protein 3 (BNIP3), kidney tissue; Beclin-1: breast tissue; microtubule-associated protein 1 light chain 3α (LC3A): brain tissue; microtubule-associated protein 1 light chain 3β (LC3B): brain tissue, p62: spleen tissue; ATP synthase: heart tissue; and glutaminase: liver tissue. Slides were counterstained with Harris hematoxylin.

**Table 2:** **Source, clone and dilution of antibodies used in this study**^**a**^

**Antibody**	**Clone**	**Dilution**	**Company**
Molecular subtype-related			
ER	SP1	1:100	Thermo Scientific, Waltham, MA, USA
PR	PgR	1:50	Dako Denmark AS, Glostrup, Denmark
HER2	Polyclonal	1:1,500	Dako Denmark AS, Glostrup, Denmark
Ki-67	MIB-1	1:150	Dako Denmark AS, Glostrup, Denmark
Glycolysis-related			
Glut-1	SPM498	1:200	Abcam, Cambridge, UK
CAIX	Polyclonal	1:100	Abcam, Cambridge, UK
MCT4	Polyclonal	1:100	Santa Cruz Biotechnology, Santa Cruz, CA, USA
Mitochondrial status-related			
BNIP3	Ana40	1:100	Abcam, Cambridge, UK
Mitochondrial metabolism-related			
ATP synthase	15H4C4	1:100	Abcam, Cambridge, UK
Glutaminase	Polyclonal	1:100	Abcam, Cambridge, UK
Autophagy-related			
Beclin-1	Polyclonal	1:100	Abcam, Cambridge, UK
LC3A	EP1528Y	1:100	Abcam, Cambridge, UK
LC3B	Polyclonal	1:100	Abcam, Cambridge, UK
p62	SQSTM1	1:100	Abcam, Cambridge, UK

^a^BNIP3, BCL2/adenovirus E1B 19-kDa interacting protein 3; CAIX, carbonic anhydrase IX; ER, estrogen receptor; Glut-1, glucose transporter 1; LC3A, microtubule-associated protein 1 light chain 3α; LC3B, microtubule-associated protein 1 light chain 3β; MCT4, monocarboxylate transporter 4; PR, progesterone receptor.

### Interpretation of immunohistochemical staining

All immunohistochemical markers were assessed by light microscopy. Pathologic parameters such as ER, PR and HER2 expression were obtained from each patient’s pathologic report. A cutoff value of 1% or more positively stained nuclei was used to define ER and PR positivity [14]. HER2 staining was analyzed according to the American Society of Clinical Oncology (ASCO)/College of American Pathologists (CAP) guidelines using the following categories: 0 = no immunostaining; 1+ = weak, incomplete membranous staining, less than 10% of tumor cells; 2+ = complete membranous staining, either uniform or weak in at least 10% of tumor cells; and 3+ = uniform intense membranous staining in at least 30% of tumor cells [15]. HER2 immunostaining was considered positive when strong (3+) membranous staining was observed, whereas cases with 0 to 1+ were regarded as negative. Cases showing 2+ HER2 expression were evaluated for HER2 amplification by fluorescence *in situ* hybridization (FISH).

Glut-1, CAIX, BNIP3, MCT4, Beclin-1, LC3A, LC3B and p62 immunohistochemical staining was evaluated on the basis of the proportion of stained cells and immunostaining intensity. The proportion of stained cells was graded 0 (negative), 1 (less than 30% positive) or 2 (more than 30% positive). Immunostaining intensity was graded as 0 (negative), 1 (weak), 2 (moderate) or 3 (strong). The scores for the proportion of stained cells and staining intensity were multiplied to provide a total score: negative (0 or 1) or positive (2 through 6). Ki-67 labeling indices (LIs) were scored by counting the number of positively stained nuclei and expressed as a percentage of total tumor cells.

### Fluorescence *in situ* hybridization analysis

Before FISH analysis, invasive tumors were examined on H&E-stained slides. FISH was subsequently performed on the confirmed tumor. FISH was performed using the PathVysion HER-2 DNA Probe Kit (Abbott Molecular, Abbott Park, IL, USA) according to the manufacturer’s instructions. *HER2* gene copy number on the slides was evaluated using an epifluorescence microscope (Olympus, Tokyo, Japan). At least 60 tumor cell nuclei in three separate regions were investigated for *HER2* and chromosome 17 signals. *HER2* gene amplification was determined according to the ASCO/CAP guidelines [15]. An absolute *HER2* gene copy number lower than 4 or a *HER2* gene/chromosome 17 (chr17) copy number ratio (HER2/chr17 ratio) less than 1.8 was considered *HER2*-negative. An absolute *HER2* copy number between 4 and 6 or a HER2/chr17 ratio between 1.8 and 2.2 was considered *HER2*-equivocal. An absolute *HER2* copy number greater than 6 or a HER2/chr17 ratio higher than 2.2 was considered *HER2*-positive.

### Tumor phenotype classification

In this study, we classified breast cancer phenotypes according to the IHC results for ER, PR, HER2 and Ki-67 LI. FISH results for HER2 were as follows [16]: luminal A type: ER- and/or PR-positive, HER2-negative Ki-67 LI less than 14%; luminal B type: (HER2-negative) ER- and/or PR-positive, HER2-negative and Ki-67 LI greater than or equal to 14% and (HER2-positive) ER- and/or PR-positive and HER2 overexpressed and/or amplified; HER2 type: ER- and PR-negative and HER2 overexpressed and/or amplified; TNBC type: ER-, PR- and HER2-negative.

### Classification of tumor metabolic subtypes

We also classified cases based on the results of immunohistochemical staining for metabolism-related proteins as follows: glycolysis type: Glut-1- and/or CAIX-positive; nonglycolysis type: Glut-1- and CAIX-negative; dysfunctional mitochondrial type: BNIP3-positive [17,18]; functional mitochondrial type: BNIP3-negative; activated autophagy type: positive for two or more markers from among Beclin-1, LC3A, LC3B and p62; and nonactivated autophagy type: positive for less than two markers from among Beclin-1, LC3A, LC3B and p62. We further classified the metabolic phenotypes of breast cancer as follows: Warburg type: tumor (glycolysis type), stroma (nonglycolysis type); reverse Warburg type: tumor (nonglycolysis type), stroma (glycolysis type); mixed type: tumor (glycolysis type), stroma (glycolysis); and null type: tumor (nonglycolysis type), stroma (non-glycolysis type).

### Laser microdissection and protein extraction from formalin-fixed, paraffin-embedded tissues

To acquire tumors and tumor stroma, laser microdissection was performed with hematoxylin-stained, uncovered slides generated with FFPE blocks (LMD 6500; Leica, Wetzlar, Germany). Five cases per molecular subtype of breast cancer were microdissected. Protein extraction from microdissected FFPE tissues was performed using the Qproteome FFPE Tissue Kit (QIAGEN, Hilden, Germany). Briefly, microdissected FFPE tissues were deparaffinized in xylene and rehydrated in a graded series of alcohol. Afterward, the samples were mixed with FFPE extraction buffer EXB Plus (100 μl per sample; QIAGEN), incubated at 100°C for 20 min, at 80°C for 2 h and then centrifuged for 15 min at 14,000 × *g* at 4°C. The protein concentrations in the supernatant were determined using the Bradford assay (Bio-Rad Laboratories, Hercules, CA, USA).

### Western blot analysis

Total protein (20 μg) from each sample was mixed with Laemmli sample buffer and heated at 100°C for 5 min. It was then loaded into individual wells, resolved by 8% SDS-PAGE and electroblotted onto nitrocellulose membranes (GE Healthcare Life Sciences, Pittsburgh, PA, USA). Membranes were blocked in 5% nonfat dry milk in Tris-buffered saline with Tween 20 (TBS-T), then incubated with antibodies to Glut-1, CAIX, ATP synthase, glutaminase, MCT-4, LC3A and p62 overnight at 4°C. The membranes were washed with TBS-T and then probed with peroxidase-conjugated goat anti-rabbit immunoglobulin G (1:2,000; Santa Cruz Biotechnology, Santa Cruz, CA, USA) for 1 h at room temperature. Washing was repeated and the membranes were developed with an enhanced chemiluminescence agent (Amersham/GE Healthcare Life Sciences, Little Chalfont, UK). Band densities were measured using TINA image software (raytest, Straubenhardt, Germany).

### Statistical analyses

Data were processed using SPSS for Windows version 12.0 software (SPSS Inc, Chicago, IL, USA). Student’s *t*-test and Fisher’s exact test were used to examine any differences in continuous and categorical variables, respectively. Significance was assumed when *P* < 0.05. Kaplan-Meier survival curves and logrank statistics were employed to evaluate time to tumor recurrence and time to survival. Multivariate regression analysis was performed using the Cox proportional hazards model.

## Results

### Patients’ characteristics according to tumor phenotype

The clinicopathologic characteristics of 740 patients, comprising 298 (40.3%) cases of luminal A, 166 (22.4%) cases of luminal B, 69 (9.3%) cases of HER2 type and 207 (28%) cases of TNBC type, are summarized in Table 3. TNBC type had the highest histologic grade, tumor stage and Ki-67 LI (*P* < 0.001, *P* = 0.002 and *P* < 0.001, respectively). In addition, HER2 and TNBC types had higher incidences of tumor recurrence and patient death than other types (*P* < 0.001).

**Table 3:** **Clinicopathologic characteristics of patients according to breast cancer phenotype**^**a**^

**Parameters**	**Total**	**Luminal A**	**Luminal B**	**HER2**	**TNBC**	***P*-value**
	**(*N *** **= 740) (%)**	**(*n *** **= 298) (%)**	**(*n *** **= 166) (%)**	**(*n *** **= 69) (%)**	**(*n *** **= 207) (%)**	
Age (years, mean ± SD)	49.7 ± 11.0	50.6 ± 10.5	48.5 ± 10.1	52.8 ± 9.8	48.4 ± 12.4	0.007
Histologic grade						<0.001
I	118 (15.9)	90 (30.2)	18 (10.8)	1 (1.4)	9 (4.3)	
II	373 (50.4)	180 (60.4)	90 (54.2)	35 (50.7)	68 (32.9)	
III	249 (33.6)	28 (9.4)	58 (34.9)	33 (47.8)	130 (62.8)	
Tumor stage						0.002
T1	358 (48.4)	166 (55.7)	86 (51.8)	31 (44.9)	75 (36.2)	
T2	367 (49.6)	125 (41.9)	78 (47.0)	37 (53.6)	127 (61.4)	
T3	15 (2.0)	7 (2.3)	2 (1.2)	1 (1.4)	5 (2.4)	
Nodal stage						0.041
N0	436 (58.9)	168 (56.4)	91 (54.8)	42 (60.9)	135 (65.2)	
N1	200 (27.0)	90 (30.2)	43 (25.9)	13 (18.8)	54 (26.1)	
N2	66 (8.9)	27 (9.1)	17 (18.5)	10 (14.5)	12 (5.8)	
N3	38 (5.1)	13 (4.4)	15 (9.0)	4 (5.8)	6 (2.9)	
Estrogen receptor status						<0.001
Negative	286 (38.6)	5 (1.7)	5 (3.0)	69 (100.0)	207 (100.0)	
Positive	454 (61.4)	293 (98.3)	161 (97.0)	0 (0.0)	0 (0.0)	
Progesterone receptor status						<0.001
Negative	371 (50.1)	50 (16.8)	46 (27.7)	69 (100.0)	207 (100.0)	
Positive	369 (49.9)	248 (83.2)	120 (72.3)	0 (0.0)	0 (0.0)	
HER2 status						<0.001
0	290 (39.2)	108 (36.2)	23 (13.9)	0 (0.0)	159 (76.8)	
1+	186 (25.1)	118 (39.6)	33 (20.0)	0 (0.0)	35 (16.9)	
2+	142 (19.2)	72 (24.2)	41 (24.7)	16 (23.2)	13 (6.3)	
3+	122 (16.5)	0 (0.0)	69 (41.6)	53 (76.8)	0 (0.0)	
Ki-67 LI (%, mean ± SD)	18.1 ± 19.2	4.7 ± 3.7	19.7 ± 12.7	19.5 ± 12.5	35.6 ± 23.7	<0.001
Tumor recurrence	69 (9.3)	15 (5.0)	12 (7.2)	11 (15.9)	31 (15.0)	<0.001
Patient’s death	67 (9.1)	14 (4.7)	11 (6.6)	12 (17.4)	30 (14.5)	<0.001
Duration of clinical follow-up (months, mean ± SD)	70.2 ± 31.7	72.7 ± 30.0	70.3 ± 30.3	67.1 ± 35.8	67.8 ± 33.8	0.291

^a^LI, labeling index; TNBC triple-negative breast cancer.

### Expression of metabolism-related proteins according to tumor phenotype

The differential expression of metabolism-related proteins according to breast cancer phenotype is summarized in Table 4. Tumor expression of Glut-1, MCT4 and LC3A was highest in TNBC and lowest in the luminal A type (*P* < 0.001). Stromal expression of CAIX and MCT4 and tumor expression of cytoplasmic p62 was highest in HER2 type and lowest in luminal A type (*P* = 0.032, *P* < 0.001 and *P* < 0.001, respectively). Tumor expression of CAIX and LC3B was highest in TNBC and lowest in luminal B type (*P* = 0.008 and *P* = 0.013, respectively). HER2 type showed the highest tumor and stromal ATP synthase expression (*P* = 0.027 and *P* < 0.001, respectively) and stromal glutaminase expression (*P* = 0.001), whereas luminal A type showed the lowest expression of those markers. Expression of stromal LC3A and tumor expression of nuclear p62 were highest in luminal A and lowest in TNBC (*P* < 0.001).

**Table 4:** **Expression of metabolism-related proteins according to breast cancer phenotype**^**a**^

**Parameters**	**Total**	**Luminal A**	**Luminal B**	**HER2**	**TNBC**	***P*-value**
	**(*N *** **= 740 ) (%)**	**(*n *** **= 298 ) (%)**	**(*n *** **= 166) (%)**	**(*n *** **= 69) (%)**	**(*n *** **= 207) (%)**	
Glut-1 in tumor						<0.001
Negative	504 (68.1)	260 (87.2)	124 (74.7)	47 (68.1)	73 (35.3)	
Positive	236 (31.9)	38 (12.8)	42 (25.3)	22 (31.9)	134 (64.7)	
Glut-1 in stroma						0.103
Negative	724 (97.8)	296 (99.3)	162 (97.6)	66 (95.7)	200 (96.6)	
Positive	16 (2.2)	2 (0.7)	4 (2.4)	3 (4.3)	7 (3.4)	
CAIX in tumor						0.008
Negative	520 (70.3)	217 (72.8)	127 (76.5)	49 (71.0)	127 (61.3)	
Positive	220 (29.7)	81 (27.2)	39 (23.5)	20 (29.0)	80 (38.6)	
CAIX in stroma						0.032
Negative	627 (84.7)	264 (88.6)	137 (82.5)	52 (75.4)	174 (84.1)	
Positive	113 (15.3)	34 (11.4)	29 (17.5)	17 (24.6)	33 (15.9)	
ATP synthase in tumor						0.027
Negative	30 (4.1)	20 (6.7)	4 (2.4)	1 (1.4)	5 (2.4)	
Positive	710 (95.9)	278 (93.3)	162 (97.6)	68 (98.6)	202 (97.6)	
ATP synthase in stroma						<0.001
Negative	570 (77.0)	256 (85.9)	112 (67.5)	38 (55.1)	164 (79.2)	
Positive	170 (23.0)	42 (14.1)	54 (32.5)	31 (44.9)	43 (20.8)	
Glutaminase in tumor						0.164
Negative	219 (29.6)	85 (28.5)	60 (36.1)	21 (30.4)	53 (25.6)	
Positive	521 (70.4)	213 (71.5)	106 (63.9)	48 (69.6)	154 (74.4)	
Glutaminase in stroma						0.001
Negative	495 (66.9)	223 (74.8)	105 (63.3)	39 (56.5)	128 (61.8)	
Positive	245 (33.1)	75 (25.2)	61 (36.7)	30 (43.5)	79 (38.2)	
BNIP3 in tumor						0.262
Negative	504 (68.1)	206 (69.1)	112 (67.5)	40 (58.0)	146 (70.5)	
Positive	236 (31.9)	92 (30.9)	54 (32.5)	29 (42.0)	61 (29.5)	
BNIP3 in stroma						0.262
Negative	700 (94.6)	281 (94.3)	159 (95.8)	62 (89.9)	198 (95.7)	
Positive	40 (5.4)	17 (5.7)	7 (4.2)	7 (10.1)	9 (4.3)	
MCT4 in tumor						<0.001
Negative	540 (73.0)	253 (84.9)	118 (71.1)	49 (71.0)	120 (58.0)	
Positive	200 (27.0)	45 (15.1)	48 (28.9)	20 (29.0)	87 (42.0)	
MCT4 in stroma						<0.001
Negative	418 (56.5)	222 (74.5)	81 (48.8)	23 (33.3)	92 (44.4)	
Positive	322 (43.5)	76 (25.5)	85 (51.2)	46 (66.7)	115 (55.6)	
Cytoplasmic Beclin-1						0.137
Negative	406 (54.9)	169 (56.7)	99 (59.6)	31 (44.9)	107 (51.7)	
Positive	334 (45.1)	129 (43.3)	67 (33.7)	38 (55.1)	100 (48.3)	
Nuclear Beclin-1						<0.001
Negative	666 (90.0)	262 (87.9)	152 (91.6)	55 (79.7)	197 (95.2)	
Positive	74 (10.0)	36 (12.1)	14 (8.4)	14 (20.3)	10 (4.8)	
LC3A in tumor						<0.001
Negative	669 (90.4)	294 (98.7)	158 (95.2)	68 (98.6)	149 (72.0)	
Positive	71 (9.6)	4 (1.3)	8 (4.8)	1 (1.4)	58 (28.0)	
LC3A in stroma						<0.001
Negative	687 (92.8)	267 (89.6)	151 (91.0)	62 (89.9)	207 (100.0)	
Positive	53 (7.2)	31 (10.4)	15 (9.0)	7 (10.1)	0 (0.0)	
LC3B in tumor						0.013
Negative	475 (64.2)	186 (62.4)	124 (74.7)	42 (60.9)	123 (59.4)	
Positive	265 (35.8)	112 (37.6)	42 (25.3)	27 (39.1)	84 (40.6)	
LC3B in stroma						0.645
Negative	688 (93.0)	277 (93.0)	151 (91.0)	65 (94.2)	195 (94.2)	
Positive	52 (7.0)	21 (7.0)	15 (9.0)	4 (5.8)	12 (5.8)	
Cytoplasmic p62 in tumor						<0.001
Negative	274 (37.0)	131 (44.0)	51 (30.7)	15 (21.7)	77 (37.2)	
Positive	466 (63.0)	167 (56.0)	115 (69.3)	54 (78.3)	130 (62.8)	
Nuclear p62 in tumor						<0.001
Negative	532 (71.9)	180 (60.4)	131 (78.9)	44 (63.8)	177 (85.5)	
Positive	208 (28.1)	118 (39.6)	35 (21.1)	25 (36.2)	30 (14.5)	
Nuclear p62 in stroma						0.876
Negative	512 (69.2)	206 (69.1)	115 (69.3)	45 (65.2)	146 (70.5)	
Positive	228 (30.8)	92 (30.9)	51 (30.7)	24 (34.8)	61 (29.5)	

^a^BNIP3, BCL2/adenovirus E1B 19-kDa interacting protein 3; CAIX, carbonic anhydrase IX; Glut-1, glucose transporter 1; LC3A, microtubule-associated protein 1 light chain 3α; LC3B, microtubule-associated protein 1 light chain 3β; MCT4, monocarboxylate transporter 4; TNBC, triple-negative breast cancer.

### Correlation between metabolism-related proteins and clinicopathologic factors

The correlation between expression of metabolism-related proteins and clinicopathologic parameters is summarized in Table 5. Tumor expression of Glut-1 was associated with higher histologic grade (*P <* 0.001), ER negativity (*P <* 0.001), higher T stage (*P <* 0.001) and higher Ki-67 LI (*P <* 0.001), whereas CAIX was associated with higher Ki-67 LI (*P <* 0.001). Stromal ATP synthase expression was associated with HER2 positivity (*P* < 0.001), and stromal glutaminase expression was associated with higher KI-67 LI (*P* = 0.021). Tumor expression of MCT4 was associated with higher histologic grade (*P <* 0.001), ER negativity (*P <* 0.001), PR negativity (*P <* 0.001), higher T stage (*P <* 0.001) and higher Ki-67 LI (*P <* 0.001). Stromal expression of MCT4 was associated with higher histologic grade (*P <* 0.001), ER negativity (*P <* 0.001), PR negativity (*P <* 0.001), HER2 positivity (*P <* 0.001) and higher Ki-67 LI (*P <* 0.001). Tumor expression of LC3A was associated with higher histologic grade (*P <* 0.001), ER negativity (*P <* 0.001), PR negativity (*P <* 0.001), HER2 negativity (*P <* 0.001) and higher Ki-67 LI (*P <* 0.001). In contrast, stromal expression of LC3A was associated with ER positivity (*P <* 0.001), PR positivity (*P* < 0.001) and lower Ki-67 LI (*P* = 0.032). Tumor expression of cytoplasmic p62 was associated with HER2 positivity (*P* < 0.001), whereas nuclear p62 was associated with lower histologic grade (*P* < 0.001), ER positivity (*P* < 0.001), PR positivity (*P* < 0.001) and lower Ki-67 LI (*P* < 0.001).

**Table 5:** **Correlations between the expression of metabolism-related proteins and clinicopathologic parameters**^**a**^

**Parameters**	**Glut-1 in tumor**			**Glut-1 in stroma**			**CAIX in tumor**			**CAIX in stroma**					
	**Negative**	**Positive**		**Negative**	**Positive**		**Negative**	**Positive**		**Negative**	**Positive**				
	**(*n *** **= 504) (%)**	**(*n *** **= 236) (%)**	***P*-value**	**(*n *** **= 724) (%)**	**(*n *** **= 16) (%)**	***P*-value**	**(*n *** **= 520) (%)**	**(*n *** **= 220) (%)**	***P*-value**	**(*n *** **= 627) (%)**	**(*n *** **= 113) (%)**	***P*-value**			
Age (years, mean ± SD)	50.5 ± 10.7	48.1 ± 11.4	0.126	49.7 ± 110.0	49.3 ± 9.0	18.14	49.7 ± 11.0	49.8 ± 11.0	19.67	49.3 ± 11.1	51.7 ± 10.3	0.840			
Histologic grade			<0.001			8.946			0.441			0.483			
I/II	392 (71.8)	99 (41.9)		482 (67.3)	9 (56.3)		359 (69.0)	132 (60.0)		427 (68.1)	64 (56.6)				
III	112 (22.2)	137 (58.1)		242 (33.4)	7 (43.7)		161 (31.0)	88 (40.0)		200 (31.9)	49 (43.4)				
ER			<0.001			0.378			0.042			1.113			
Negative	128 (25.4)	158 (66.9)		275 (38.0)	11 (68.8)		182 (35.0)	104 (47.3)		233 (37.2)	53 (46.9)				
Positive	376 (74.6)	78 (33.1)		449 (62.0)	5 (31.2)		338 (65.0)	116 (52.7)		394 (62.8)	60 (53.1)				
PR			<0.001			4.305			10.92			8.715			
Negative	190 (37.7)	182 (77.1)		361 (49.9)	11 (68.8)		257 (49.4)	115 (52.3)		311 (49.6)	61 (54.0)				
Positive	314 (62.3)	54 (22.9)		363 (50.1)	5 (31.2)		263 (50.6)	105 (47.7)		316 (50.4)	52 (46.0)				
HER2			0.714			6.741			0.294			1.260			
Negative	386 (76.6)	197 (83.5)		572 (79.0)	11 (68.8)		397 (76.3)	186 (84.5)		502 (80.1)	81 (71.7)				
Positive	118 (23.4)	39 (16.5)		152 (21.0)	5 (31.2)		123 (23.7)	34 (15.5)		125 (19.9)	32 (28.3)				
Tumor stage			<0.001			18.81			19.65			3.192			
T1	270 (53.6)	88 (37.3)		350 (48.3)	8 (50.0)		251 (48.3)	107 (48.6)		296 (47.2)	62 (54.9)				
T2/T3	234 (46.4)	148 (62.7)		374 (51.7)	8 (50.0)		269 (51.7)	113 (51.4)		331 (52.8)	51 (45.1)				
Nodal stage			1.932			16.12			15.62			15.87			
N0	286 (56.7)	150 (63.6)		426 (58.8)	10 (62.5)		304 (58.5)	132 (60.0)		371 (59.2)	65 (57.5)				
N1/N2/N3	218 (43.3)	86 (36.4)		298 (41.2)	6 (37.5)		216 (41.5)	88 (40.0)		256 (40.8)	48 (42.5)				
Ki-67 LI (%, mean ± SD)	12.7 ± 14.9	29.6 ± 22.1	<0.001	18.0 ± 19.3	22.5 ± 14.7	7.497	16.1 ± 17.5	22.7 ± 22.1	<0.001	17.8 ± 19.7	19.5 ± 16.7	8.505			
Tumor recurrence			0.210			8.106			18.62			17.85			
Absent	467 (92.7)	204 (86.4)		655 (90.5)	16 (100.0)		471 (90.6)	200 (90.9)		568 (90.6)	103 (91.2)				
Present	37 (7.3)	32 (13.6)		69 (9.5)	0 (0.0)		49 (9.4)	20 (9.1)		59 (9.4)	10 (9.8)				
Death			0.420			8.085			11.76			15.22			
Survival	467 (92.7)	206 (87.3)		657 (90.7)	16 (100.0)		475 (91.3)	198 (90.0)		571 (91.1)	102 (90.3)				
Death	37 (7.3)	30 (12.7)		67 (9.3)	0 (0.0)		45 (8.7)	22 (10.0)		56 (8.9)	11 (9.7)				
**Parameters**	**ATP synthase in tumor**			**ATP synthase in stroma**			**Glutaminase in tumor**			**Glutaminase in stroma**					
	**Negative**	**Positive**		**Negative**	**Positive**		**Negative**	**Positive**		**Negative**	**Positive**				
	**(*n *** **= 30) (%)**	**(*n *** **= 710) (%)**	***P*-value**	**(*n *** **= 570) (%)**	**(*n *** **= 170) (%)**	***P*-value**	**(*n *** **= 219) (%)**	**(*n *** **= 521) (%)**	***P*-value**	**(*n *** **= 495) (%)**	**(*n *** **= 245) (%)**	***P*-value**			
Age (years, mean ± SD)	46.7 ± 9.3	49.9 ± 11.0	2.478	49.5 ± 11.2	50.4 ± 10.0	7.539	49.2 ± 11.6	49.9 ± 10.7	11.90	49.5 ± 11.1	50.4 ± 10.7	5.124			
Histologic grade			11.63			0.252			16.77			0.168			
I/II	22 (733)	469 (66.1)		392 (68.8)	99 (58.2)		147 (67.1)	344 (66.0)		345 (69.7)	146 (59.6)				
III	8 (26.7)	241 (33.9)		178 (31.2)	71 (41.8)		72 (32.9)	177 (34.0)		150 (30.3)	99 (40.4)				
ER			1.827			1.533			4.536			0.126			
Negative	7 (23.3)	279 (39.3)		210 (36.8)	76 (44.7)		77 (35.2)	209 (40.1)		174 (35.2)	112 (45.7)				
Positive	23 (76.7)	431 (60.7)		360 (63.2)	94 (55.3)		142 (64.8)	312 (59.9)		321 (64.8)	133 (54.3)				
PR			0.189			0.609			4.158			0.735			
Negative	8 (26.7)	364 (51.3)		274 (48.1)	98 (57.6)		102 (46.6)	270 (51.8)		235 (47.5)	137 (55.9)				
Positive	22 (73.3)	346 (48.7)		296 (51.9)	72 (42.4)		117 (53.4)	251 (48.2)		260 (52.5)	108 (44.1)				
HER2			3.570			<0.001			13.08			3.822			
Negative	27 (90.0)	556 (78.3)		472 (82.8)	111 (65.3)		170 (77.6)	413 (79.3)		397 (80.2)	186 (75.9)				
Positive	3 (10.0)	154 (21.7)		98 (17.2)	59 (34.7)		49 (22.4)	108 (20.7)		98 (19.8)	59 (24.1)				
Tumor stage			7.539			21.00			7.875			18.39			
T1	12 (40.0)	346 (48.7)		276 (48.4)	82 (48.2)		100 (45.7)	258 (49.5)		238 (48.1)	120 (49.0)				
T2/T3	18 (60.0)	364 (51.3)		294 (51.6)	88 (51.8)		119 (54.3)	263 (50.5)		257 (51.9)	125 (51.0)				
Nodal stage			7.266			6.027			7.728			8.967			
N0	15 (50.0)	421 (59.3)		342 (60.0)	94 (55.3)		135 (61.6)	301 (57.8)		297 (60.0)	139 (56.7)				
N1/N2/N3	15 (50.0)	289 (40.7)		228 (40.0)	76 (44.7)		84 (38.4)	220 (42.2)		198 (40.0)	106 (43.3)				
Ki-67 LI (%, mean ± SD)	9.8 ± 11.7	18.4 ± 19.4	0.336	17.3 ± 19.7	20.8 ± 17.5	0.798	18.6 ± 20.0	17.8 ± 18.9	13.50	15.6 ± 17.2	22.9 ± 22.0	0.021			
Tumor recurrence			10.79			21.00			7.014			10.56			
Absent	26 (86.7)	645 (90.8)		517 (90.7)	154 (90.6)		195 (89.0)	476 (91.4)		446 (90.1)	225 (91.8)				
Present	4 (13.3)	65 (9.2)		53 (9.3)	16 (9.4)		24 (11.0)	45 (8.6)		49 (9.9)	20 (8.2)				
Death			15.68			13.60			3.381			8.757			
Survival	27 (90.0)	646 (91.0)		520 (91.2)	153 (90.0)		194 (88.6)	479 (91.9)		447 (90.3)	226 (92.2)				
Death	3 (10.0)	64 (9.0)		50 (8.8)	17 (10.0)		25 (11.4)	42 (8.1)		48 (9.7)	19 (7.8)				
**Parameters**	**BNIP3 in tumor**			**BNIP3 in stroma**			**MCT4 in tumor**			**MCT4 in stroma**					
	**Negative**	**Positive**		**Negative**	**Positive**		**Negative**	**Positive**		**Negative**	**Positive**				
	**(*n *** **=504) (%)**	**(*n *** **= 236) (%)**	***P*-value**	**(*n *** **= 700) (%)**	**(*n *** **= 40) (%)**	***P*-value**	**(*n *** **= 540) (%)**	**(*n *** **= 200) (%)**	***P*-value**	**(*n *** **= 418) (%)**	**(*n *** **= 322) (%)**	***P*-value**			
Age (years, mean ± SD)	48.9 ± 10.9	51.6 ± 11.0	0.042	49.5 ± 10.8	53.2 ± 12.7	0.882	49.8 ± 11.1	49.6 ± 10.6	17.91	49.6 ± 11.0	49.5 ± 10.9	14.80			
Histologic grade			5.859			15.37			<0.001			<0.001			
I/II	341 (67.7)	150 (63.6)		463 (66.1)	28 (70.0)		386 (71.5)	105 (52.5)		314 (75.1)	177 (55.0)				
III	163 (32.3)	86 (36.4)		237 (33.9)	12 (30.0)		154 (28.5)	95 (47.5)		104 (24.9)	145 (45.0)				
ER			14.40			12.99			<0.001			<0.001			
Negative	192 (38.1)	94 (39.8)		269 (38.4)	17 (42.5)		174 (32.2)	112 (56.0)		123 (29.4)	163 (50.6)				
Positive	312 (61.9)	142 (60.2)		431 (61.6)	23 (57.5)		366 (67.8)	88 (44.0)		295 (70.6)	159 (49.4)				
PR			17.07			8.757			<0.001			<0.001			
Negative	255 (50.6)	117 (49.6)		349 (49.9)	23 (57.5)		232 (43.0)	140 (70.0)		179 (42.8)	193 (60.0)				
Positive	249 (49.4)	119 (50.4)		351 (50.1)	17 (42.5)		308 (57.0)	60 (30.0)		239 (57.2)	129 (40.0)				
HER2			4.410			6.762			11.42			<0.001			
Negative	404 (80.2)	179 (75.8)		554 (79.1)	29 (72.5)		422 (78.1)	161 (80.5)		356 (85.2)	227 (70.5)				
Positive	100 (19.8)	57 (24.2)		146 (20.9)	11 (27.5)		118 (21.9)	39 (19.5)		62 (14.8)	95 (29.5)				
Tumor stage			1.449			15.66			<0.001			7.854			
T1	232 (46.0)	126 (53.4)		340 (48.6)	18 (45.0)		283 (52.4)	75 (37.5)		196 (46.9)	162 (50.3)				
T2/T3	272 (54.0)	110 (46.6)		360 (51.4)	22 (55.0)		257 (47.6)	125 (62.5)		222 (53.1)	160 (49.7)				
Nodal stage			6.237			0.630			18.20			7.686			
N0	290 (57.5)	146 (61.9)		419 (59.9)	17 (42.5)		317 (58.7)	119 (59.5)		240 (57.4)	196 (60.9)				
N1/N2/N3	214 (42.5)	90 (38.1)		281 (40.1)	23 (57.5)		223 (41.3)	81 (40.5)		178 (42.6)	126 (39.1)				
Ki-67 LI (%, mean ± SD)	18.9 ± 20.9	16.2 ± 15.0	1.680	18.3 ± 19.5	14.6 ± 13.7	5.208	15.3 ± 17.8	25.6 ± 21.0	<0.001	13.2 ± 16.5	24.3 ± 20.7	<0.001			
Tumor recurrence			0.021			3.444			14.11			11.00			
Absent	445 (88.3)	226 (95.8)		632 (90.3)	39 (97.5)		491 (90.9)	180 (90.0)		376 (90.0)	295 (91.6)				
Present	59 (11.7)	10 (4.2)		68 (9.7)	1 (2.5)		49 (9.1)	20 (10.0)		42 (10.0)	27 (8.4)				
Death			5.712			15.22			11.90			2.562			
Survival	454 (90.1)	219 (92.8)		636 (90.9)	37 (92.5)		493 (91.3)	180 (90.0)		374 (89.5)	299 (92.9)				
Death	50 (9.9)	17 (7.2)		64 (9.1)	3 (7.5)		47 (8.7)	20 (10.0)		44 (10.5)	23 (7.1)				
**Parameters**	**Cytoplasmic Beclin-1**			**Nuclear Beclin-1**			**LC3A in tumor**			**LC3A in stroma**					
	**Negative**	**Positive**		**Negative**	**Positive**		**Negative**	**Positive**		**Negative**	**Positive**				
	**(*n *** **= 406) (%)**	**(*n *** **= 334) (%)**	***P*-value**	**(*n *** **= 666) (%)**	**(*n *** **= 74) (%)**	***P*-value**	**(*n *** **= 669) (%)**	**(*n *** **= 71) (%)**	***P*-value**	**(*n *** **= 687) (%)**	**(*n *** **= 53) (%)**	***P*-value**			
Age (years, mean ± SD)	48.6 ± 10.5	51.1 ± 11.4	0.042	49.6 ± 11.1	50.8 ± 9.9	8.316	50.2 ± 11.0	45.6 ± 9.8	0.021	49.7 ± 11.0	49.6 ± 9.2	19.65			
Histologic grade			14.61			<0.001			<0.001			1.029			
I/II	272 (67.0)	219 (65.6)		427 (64.1)	64 (86.5)		470 (70.3)	21 (29.6)		449 (65.4)	42 (79.2)				
III	134 (33.0)	115 (34.4)		239 (35.9)	10 (13.5)		199 (29.7)	50 (70.4)		238 (34.6)	11 (20.8)				
ER			6.090			8.022			<0.001			<0.001			
Negative	143 (35.2)	143 (42.8)		261 (39.2)	25 (33.8)		226 (33.8)	60 (84.5)		278 (40.5)	8 (15.1)				
Positive	263 (64.8)	191 (57.2)		405 (60.8)	49 (66.2)		443 (66.2)	11 (15.5)		409 (59.5)	45 (84.9)				
PR			19.76			1.827			<0.001			<0.001			
Negative	205 (50.5)	167 (50.0)		342 (51.4)	30 (40.5)		309 (46.2)	63 (88.7)		360 (52.4)	12 (22.6)				
Positive	201 (49.5)	167 (50.0)		324 (48.6)	44 (59.5)		360 (53.8)	8 (11.3)		327 (47.6)	41 (77.4)				
HER2			12.36			0.336			<0.001			8.022			
Negative	323 (79.6)	260 (77.8)		533 (80.0)	50 (67.6)		515 (76.9)	68 (95.8)		544 (79.2)	39 (73.6)				
Positive	83 (20.4)	74 (22.2)		133 (20.0)	24 (32.4)		154 (23.1)	3 (4.2)		143 (20.8)	14 (26.4)				
Tumor stage			0.042			6.888			16.86			0.210			
T1	175 (43.1)	183 (54.8)		318 (47.7)	40 (54.1)		325 (48.6)	33 (46.5)		323 (47.0)	35 (66.0)				
T2/T3	231 (56.9)	151 (45.2)		348 (52.3)	34 (45.9)		344 (51.4)	38 (53.5)		364 (53.0)	18 (34.0)				
Nodal stage			13.71			3.612			6.552			16.23			
N0	236 (58.1)	200 (59.9)		398 (59.8)	38 (51.4)		390 (58.3)	46 (64.8)		406 (59.1)	30 (56.6)				
N1/N2/N3	170 (41.9)	134 (40.1)		268 (40.2)	36 (48.6)		279 (41.7)	25 (35.2)		281 (40.9)	23 (43.4)				
Ki-67 LI (%, mean ± SD)	17.8 ± 19.4	18.3 ± 19.1	0.042	19.0 ± 19.8	9.5 ± 10.0	<0.001	15.7 ± 17.2	39.6 ± 23.1	<0.001	18.7 ± 19.7	10.4 ± 9.6	0.032			
Tumor recurrence			0.882			2.877			4.053			16.98			
Absent	360 (88.7)	311 (93.1)		600 (90.1)	71 (95.9)		610 (91.2)	61 (85.9)		622 (90.5)	49 (92.5)				
Present	46 (11.3)	23 (6.9)		66 (9.9)	3 (4.1)		59 (8.8)	10 (14.1)		65 (9.5)	4 (7.5)				
Death			16.75			0.189			10.75			9.765			
Survival	368 (90.6)	305 (91.3)		600 (90.1)	73 (98.6)		610 (91.2)	63 (88.7)		623 (90.7)	50 (94.3)				
Death	38 (9.4)	29 (8.7)		66 (9.9)	1 (1.4)		59 (8.8)	8 (11.3)		64 (9.3)	3 (5.7)				
**Parameters**	**LC3B in tumor**			**LC3B in stroma**			**Cytoplasmic p62 in tumor**			**Nuclear p62 in tumor**			**Nuclear p62 in stroma**		
	**Negative**	**Positive**		**Negative**	**Positive**		**Negative**	**Positive**		**Negative**	**Positive**		**Negative**	**Positive**	
	**(*n *** **= 475) (%)**	**(n = 265) (%)**	***P*-value**	**(*n *** **= 688) (%)**	**(*n *** **= 52) (%)**	***P*-value**	**(*n *** **= 274) (%)**	**(*n *** **= 466) (%)**	***P*-value**	**(*n *** **= 532) (%)**	**(*n *** **= 208) (%)**	***P*-value**	**(*n *** **= 512) (%)**	**(*n *** **= 228) (%)**	***P*-value**
Age	49.4 ± 10.4	50.4 ± 12.0	4.599	49.6 ± 11.0	51.1 ± 10.6	15.77	49.4 ± 10.3	49.9 ± 11.4	10.35	49.4 ± 10.9	50.6 ± 11.2	3.717	49.5 ± 11.2	50.3 ± 10.4	6.741
(years, mean ± SD)															
Histologic grade			3.528			9.450			0.210			<0.001			18.18
I/II	324 (68.2)	167 (63.0)		459 (66.7)	32 (61.5)		198 (72.3)	293 (62.9)		322 (60.5)	169 (81.3)		341 (66.6)	150 (65.8)	
III	151 (31.8)	98 (37.0)		229 (33.3)	20 (38.5)		76 (27.7)	173 (37.1)		210 (39.5)	39 (18.8)		171 (33.4)	78 (34.2)	
ER			6.279			7.980			2.877			<0.001			14.34
Negative	173 (36.4)	113 (42.6)		269 (39.1)	17 (32.7)		96 (35.0)	190 (40.8)		227 (42.7)	59 (28.4)		195 (38.1)	91 (39.9)	
Positive	302 (63.6)	152 (57.4)		419 (60.9)	35 (67.3)		178 (65.0)	276 (59.2)		305 (57.3)	149 (71.6)		317 (61.9)	137 (60.1)	
PR			10.29			5.292			0.105			<0.001			17.05
Negative	234 (49.3)	138 (52.1)		350 (50.9)	22 (42.3)		119 (43.4)	253 (54.3)		293 (55.1)	79 (38.0)		259 (50.6)	113 (49.6)	
Positive	241 (50.7)	127 (47.9)		338 (49.1)	30 (57.7)		155 (56.6)	213 (45.7)		239 (44.9)	129 (62.0)		253 (49.4)	115 (50.4)	
HER2			12.07			12.55			<0.001			16.04			13.16
Negative	371 (78.1)	212 (80.0)		540 (78.5)	43 (82.7)		238 (86.9)	345 (74.0)		421 (79.1)	162 (77.9)		406 (79.3)	177 (34.6)	
Positive	104 (21.9)	53 (20.0)		148 (21.5)	9 (17.3)		36 (13.1)	121 (26.0)		111 (20.9)	46 (22.1)		106 (20.7)	51 (22.4)	
Tumor stage			0.357			14.00			3.570			0.189			0.189
T1	214 (45.1)	144 (54.3)		331 (48.1)	27 (51.9)		142 (51.8)	216 (46.4)		241 (45.3)	117 (56.2)		231 (45.1)	127 (55.7)	
T2/T3	261 (54.9)	121 (45.7)		357 (51.9)	25 (48.1)		132 (48.2)	250 (53.6)		291 (54.7)	91 (43.8)		281 (54.9)	101 (44.3)	
Nodal stage			6.531			13.90			3.969			5.901			10.87
N0	273 (57.5)	163 (61.5)		407 (59.2)	29 (55.8)		170 (62.0)	266 (57.1)		320 (60.2)	116 (55.8)		306 (59.8)	130 (57.0)	
N1/N2/N3	202 (42.5)	102 (38.5)		281 (40.8)	23 (44.2)		104 (38.0)	200 (42.9)		212 (39.8)	92 (44.2)		206 (40.2)	98 (43.0)	
Ki-67 LI (%, mean ± SD)	18.2 ± 19.8	17.8 ± 18.2	16.10	18.4 ± 19.4	18.9 ± 17.6	15.77	16.0 ± 19.2	19.3 ± 19.2	0567	21.4 ± 20.8	9.5 ± 10.6	<0.001	18.5 ± 19.7	17.0 ± 18.1	7.161
Tumor recurrence			18.81			9.765			18.83			3.339			19.80
Absent	430 (90.5)	241 (90.9)		622 (90.4)	49 (94.2)		248 (90.5)	423 (90.8)		477 (89.7)	194 (93.3)		464 (90.6)	207 (90.8)	
Present	45 (9.5)	24 (9.1)		66 (9.6)	3 (5.8)		26 (9.5)	43 (9.2)		55 (10.3)	14 (6.7)		48 (9.4)	21 (9.2)	
Death			8.925			12.89			14.53			10.03			14.28
Survival	435 (91.6)	238 (89.8)		624 (90.7)	49 (94.2)		251 (91.6)	422 (90.6)		481 (90.4)	192 (92.3)		467 (91.2)	206 (90.3)	
Death	40 (8.4)	27 (10.2)		64 (9.3)	3 (5.8)		23 (8.4)	44 (9.4)		51 (9.6)	16 (7.7)		45 (8.8)	22 (9.7)	

^a^*P*-values are corrected for multiple testing using the Bonferroni correction. BNIP3, BCL2/adenovirus E1B 19-kDa interacting protein 3; CAIX, carbonic anhydrase IX; Glut-1, glucose transporter 1; LC3A, microtubule-associated protein 1 light chain 3α; LC3B, microtubule-associated protein 1 light chain 3β; LI, labeling index; MCT4, monocarboxylate transporter 4; TNBC, triple-negative breast cancer.

### Correlation between tumor metabolic phenotype and clinicopathologic factors

**Figure 1:**
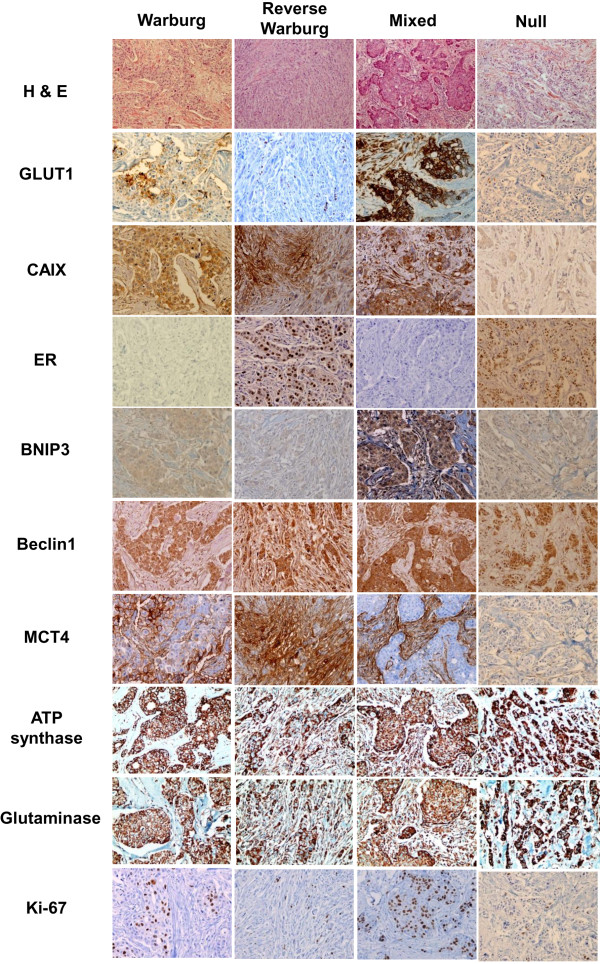
**Histologic and immunohistochemical features according to metabolic phenotypes of breast cancer.** The Warburg and mixed types show high histologic grade, estrogen receptor (ER) negativity and high Ki-67 labeling index (LI). In contrast, the reverse Warburg and null types show low histologic grade, ER positivity and low Ki-67 LI. ATP synthase and glutaminase were high in the reverse Warburg and mixed types and low in the Warburg and null types (*P* < 0.001). BNIP3, BCL2/adenovirus E1B 19-kDa interacting protein 3; CAIX, carbonic anhydrase IX; GLUT1, glucose transporter 1; H & E, hematoxylin and eosin; MCT4, monocarboxylate transporter 4.

The correlation between the metabolic phenotype of breast cancer and clinicopathologic parameters is summarized in Table 6 and Figure 1. The metabolic phenotype was the Warburg type (*n* = 298, 40.3%), the null type (*n* = 326, 44.0%), the mixed type (*n* = 62, 8.4%) and the reverse Warburg type (*n* = 54, 7.3%). Histologic grade was highest in the mixed type and lowest in the null type (*P* < 0.001). The mixed type had the highest percentage of ER and PR negativity, and the null type had the highest percentage of ER and PR positivity (*P* < 0.001). The Warburg type had the highest percentage of negative HER2 status (*P* = 0.006). The Warburg and mixed types comprised the highest percentage of TNBC, and the reverse Warburg and null types comprised the highest percentage of luminal A types (*P* < 0.001). Stromal expression of ATP synthase and glutaminase was high in the reverse Warburg and mixed types and low in the Warburg and null types (*P* < 0.001). For the status of tumor autophagy, the mixed type had the highest percentage of activated tumor autophagy and the null type had the highest percentage of nonactivated tumor autophagy (*P* < 0.001). For the status of stromal autophagy, the reverse Warburg and mixed types had a higher percentage of activation than other types (*P* < 0.001). Tumor expression of MCT4 was highest in the Warburg type and lowest in the null type (*P* < 0.001), whereas stromal expression of MCT4 was highest in the mixed type and lowest in the null type (*P* < 0.001). Ki-67 LI was highest in the mixed type and lowest in the null type (*P* < 0.001).

**Table 6:** **Clinicopathologic characteristics of patients according to metabolic phenotype**^**a**^

**Parameters**	**Warburg type**	**Reverse Warburg**	**Mixed type**	**Null type**	***P*-value**
	**(*n *** **= 298) (%)**	**type (*n *** **= 54) (%)**	**(*n *** **= 62) (%)**	**(*n *** **= 326) (%)**	
Age (years, mean ± SD)	48.5 ± 11.7	52.0 ± 10.2	51.3 ± 10.2	50.1 ± 10.5	0.052
Histologic grade					<0.001
I/II	169 (56.7)	41 (75.9)	23 (37.0)	258 (79.1)	
III	129 (43.3)	13 (24.1)	39 (72.2)	68 (20.9)	
Tumor stage					0.017
T1	123 (41.3)	29 (53.7)	34 (54.8)	172 (52.8)	
T2/T3	175 (58.7)	25 (46.3)	28 (45.2)	154 (47.2)	
Nodal stage					0.457
N0	177 (59.3)	27 (50.0)	40 (64.5)	192 (58.9)	
N1/N2/N3	121 (40.6)	27 (50.0)	22 (35.5)	134 (41.1)	
Estrogen receptor status					<0.001
Negative	152 (51.0)	15 (27.8)	39 (62.9)	80 (24.5)	
Positive	146 (49.0)	39 (72.2)	23 (37.1)	246 (75.5)	
Progesterone receptor status					<0.001
Negative	181 (60.7)	22 (40.7)	40 (64.5)	129 (39.6)	
Positive	117 (39.3)	32 (59.3)	22 (35.5)	197 (60.4)	
HER2 status					0.006
Negative	252 (84.6)	36 (66.7)	47 (75.8)	248 (76.1)	
Positive	46 (15.4)	18 (33.3)	15 (24.2)	78 (23.9)	
Molecular subtype					<0.001
Luminal A	91 (30.5)	22 (40.7)	12 (19.4)	173 (53.1)	
Luminal B	58 (19.5)	18 (33.3)	13 (21.0)	77 (23.6)	
HER2	22 (7.4)	7 (13.0)	10 (16.1)	30 (9.2)	
Triple-negative	127 (42.6)	7 (13.0)	27 (43.5)	46 (14.1)	
ATP synthase in tumor					0.178
Negative	8 (2.7)	1 (1.9)	2 (3.2)	19 (5.8)	
Positive	290 (97.3)	53 (98.1)	60 (96.8)	307 (94.2)	
ATP synthase in stroma					<0.001
Negative	247 (82.9)	29 (53.7)	33 (53.2)	261 (80.1)	
Positive	51 (17.1)	25 (46.3)	29 (46.8)	65 (19.9)	
Glutaminase in tumor					0.512
Negative	84 (28.2)	13 (24.1)	17 (27.4)	105 (32.2)	
Positive	214 (71.8)	41 (75.9)	45 (72.6)	221 (67.8)	
Glutaminase in stroma					<0.001
Negative	206 (69.1)	20 (37.0)	29 (46.8)	240 (73.6)	
Positive	92 (30.9)	34 (63.0)	33 (53.2)	86 (26.4)	
Tumor mitochondrial status					0.217
Dysfunctional	94 (31.5)	20 (37.0)	26 (41.9)	96 (29.4)	
Functional	204 (68.5)	34 (63.0)	36 (58.1)	230 (70.6)	
Stroma mitochondrial status					0.055
Dysfunctional	13 (4.4)	3 (5.6)	8 (12.9)	16 (4.9)	
Functional	285 (95.6)	51 (94.4)	54 (87.1)	310 (95.1)	
Tumor autophagy status					<0.001
Activated	168 (56.4)	28 (51.9)	45 (72.6)	117 (35.9)	
Nonactivated	130 (43.6)	26 (48.1)	17 (27.4)	209 (64.1)	
Stroma autophagy status					<0.001
Activated	9 (3.0)	11 (20.4)	13 (21.0)	21 (6.4)	
Nonactivated	289 (97.0)	43 (79.6)	49 (79.0)	305 (93.6)	
MCT4 in tumor					<0.001
Negative	180 (60.4)	38 (70.4)	40 (64.5)	282 (86.5)	
Positive	118 (39.6)	16 (29.6)	22 (35.5)	44 (13.5)	
MCT4 in stroma					<0.001
Negative	157 (52.7)	22 (40.7)	20 (32.3)	219 (67.2)	
Positive	141 (47.3)	32 (59.3)	42 (67.7)	107 (32.8)	
Ki-67 LI (%, mean ± SD)	24.6 ± 22.5	13.2 ± 11.1	25.2 ± 18.6	11.5 ± 14.1	<0.001
Tumor recurrence	38 (12.8)	6 (11.1)	4 (6.5)	21 (6.4)	0.043
Patient’s death	36 (12.1)	5 (9.3)	6 (9.7)	20 (6.1)	0.081

^a^LI, labeling index; MCT4, monocarboxylate transporter 4.

### Impact of metabolism-related proteins on patient prognosis

**Figure 2:**
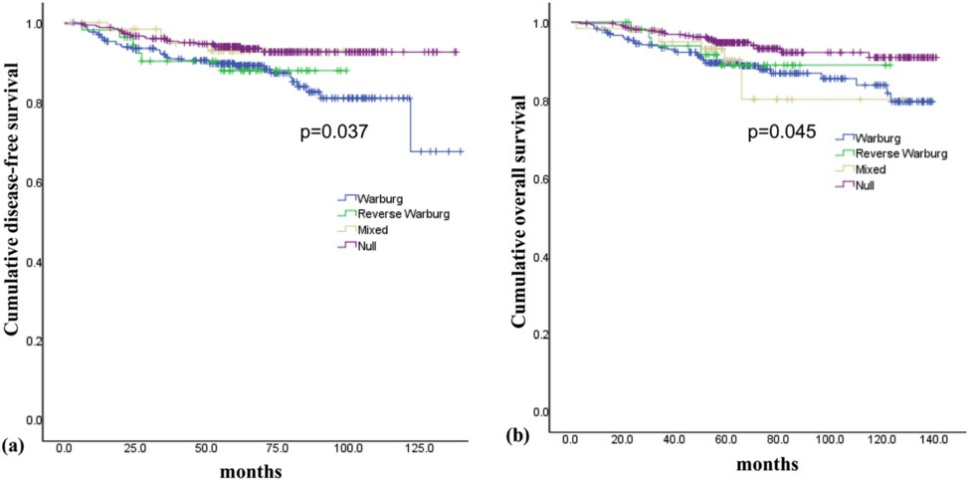
Disease-free survival (a) and overall survival curves (b) according to the metabolic phenotypes of breast cancer.

The results of univariate analysis on the correlation between metabolism-related proteins and the clinicopathologic parameters of patients are summarized in Table 7. Shorter disease-free survival (DFS) was associated with Glut-1 positivity (*P* = 0.010), BNIP3 negativity, tumor phenotype (HER2 and TNBC; *P* < 0.001) and tumor metabolic type (reverse Warburg type; *P* = 0.037) (Figure 2). Shorter overall survival (OS) was associated with Glut-1 positivity (*P* = 0.023), tumor phenotype (HER2 and TNBC; *P* < 0.001) and tumor metabolic type (mixed type; *P* = 0.045) (Figure 2). Prognostic factors evaluated by multivariate Cox analysis included histologic grade, T stage, N stage, ER status, PR status, HER2 status, tumor phenotype, tumor metabolic phenotype and tumor expression of Glut-1. The results showed that the independent factors associated with shorter DFS were ER negativity (odds ratio (OR) = 2.7, 95% CI = 1.7 to 4.5; *P* < 0.001), N stage (N0 vs. N1/2/3, OR = 2.3, 95% CI = 1.4 to 3.8; *P* = 0.001) and T stage (T1 vs. T2/3, OR = 2.4, 95% CI = 1.3 to 4.4; *P* = 0.002) and those associated with shorter OS were ER negativity (OR = 3.3, 95% CI = 2.0 to 5.5; *P* < 0.001) and N stage (N0 vs. N1/2/3, OR = 2.3, 95% CI = 1.4 to 3.8; *P* = 0.001).

**Table 7:** **Univariate analysis of the expression of metabolism-related proteins in breast cancers and disease-free survival or overall survival by logrank test**^**a**^

**Immunohistochemical factors**	**Number of patients/ recurrence/death**	**Disease-free survival**		**Overall survival**	
		**Mean survival(95% CI), months**	***P*-value**	**Mean survival (95% CI), months**	***P*-value**
Glut-1 in tumor			0.010		0.023
Negative	504/37/37	128 (125 to 131)		131 (128 to 134)	
Positive	236/32/30	119 (112 to 126)		123 (118 to 128)	
Glut-1 in stroma			n/a		n/a
Negative	724/69/67	n/a		n/a	
Positive	16/0/0	n/a		n/a	
CAIX in tumor			0.740		0.222
Negative	520/49/45	126 (122 to 130)		130 (127 to 132)	
Positive	220/20/22	108 (102 to 113)		123 (117 to 130)	
CAIX in stroma			0.927		0.496
Negative	627/59/56	125 (122 to 129)		129 (126 to 132)	
Positive	113/10/11	103 (98 to 108)		116 (109 to 123)	
ATP synthase in tumor			0.506		0.936
Negative	30/4/3	102 (90 to 114)		129 (117 to 141)	
Positive	710/65/64	125 (122 to 129)		128 (126 to 131)	
ATP synthase in stroma			0.783		0.398
Negative	570/53/50	125 (121 to 129)		129 (126 to 132)	
Positive	170/16/17	118 (112 to 124)		122 (115 to 128)	
Glutaminase in tumor			0.323		0.164
Negative	219/24/25	123 (117 to 128)		126 (120 to 131)	
Positive	521/45/42	126 (122 to 130)		130 (127 to 133)	
Glutaminase in stroma			0.554		0.596
Negative	495/49/48	123 (119 to 128)		128 (125 to 131)	
Positive	245/20/19	127 (121 to 132)		128 (124 to 133)	
BNIP3 in tumor			0.004		0.426
Negative	504/59/50	123 (119 to 127)		128 (124 to 131)	
Positive	236/10/17	123 (119 to 127)		131 (126 to 135)	
BNIP3 in stroma			0.191		0.973
Negative	700/68/64	125 (121 to 128)		128 (126 to 131)	
Positive	40/1/3	116 (111 to 121)		121 (112 to 129)	
MCT4 in tumor			0.550		0.451
Negative	540/49/47	125 (121 to 129)		129 (126 to 132)	
Positive	200/20/20	116 (111 to 121)		126 (120 to 131)	
MCT4 in stroma			0.673		0.262
Negative	418/42/44	123 (118 to 127)		127 (123 to 131)	
Positive	322/27/23	128 (124 to 132)		130 (126 to 133)	
Cytoplasmic beclin-1			0.169		0.566
Negative	406/46/38	124 (119 to 128)		129 (126 to 132)	
Positive	334/23/29	121 (118 to 124)		126 (123 to 130)	
Nuclear beclin-1			0.157		0.031
Negative	666/66/66	125 (121 to 128)		128 (125 to 131)	
Positive	74/3/1	111 (106 to 115)		136 (132 to 139)	
LC3A in tumor			0.085		0.299
Negative	669/59/59	126 (122 to 129)		129 (126 to 132)	
Positive	71/10/8	113 (103 to 122)		124 (115 to 133)	
LC3A in stroma			0.801		0.541
Negative	687/65/64	125 (122 to 129)		128 (126 to 131)	
Positive	53/4/3	65 (62 to 68)		66 (64 to 68)	
LC3B in tumor			0.990		0.271
Negative	475/45/40	125 (121 to 130)		130 (127 to 133)	
Positive	265/24/27	118 (113 to 123)		125 (120 to 130)	
LC3B in stroma			0.481		0.565
Negative	688/66/64	125 (122 to 129)		128 (126 to 131)	
Positive	52/3/3	63 (60 to 66)		64 (62 to 66)	
Cytoplasmic p62 in tumor			0.958		0.528
Negative	274/26/23	121 (112 to 129)		129 (125 to 133)	
Positive	466/43/44	126 (122 to 130)		128 (125 to 131)	
Nuclear p62 in tumor			0.210		0.646
Negative	532/55/51	125 (122 to 129)		128 (125 to 131)	
Positive	208/14/16	117 (110 to 124)		128 (122 to 133)	
Nuclear p62 in stroma			0.720		0.387
Negative	512/48/45	126 (122 to 130)		129 (126 to 132)	
Positive	228/21/22	104 (99 to 109)		124 (118 to 130)	
Tumor phenotype			<0.001		<0.001
Luminal A	298/15/14	130 (126 to 133)		134 (131 to 137)	
Luminal B	166/12/11	129 (124 to 134)		130 (124 to 135)	
HER2	69/11/12	111 (100 to 121)		119 (108 to 130)	
TNBC	207/31/30	116 (109 to 124)		120 (114 to 126)	
Metabolic status			0.037		0.045
Warburg type	298/38/36	119 (112 to 126)		124 (119 to 128)	
Reverse Warburg type	54/6/5	90 (83 to 96)		113 (106 to 121)	
Mixed type	62/4/6	105 (100 to 111)		112 (99 to 126)	
Null type	326/21/20	129 (126 to 133)		132 (129 to 136)	

^a^BNIP3, BCL2/adenovirus E1B 19-kDa interacting protein 3; CAIX, carbonic anhydrase IX; Glut-1, glucose transporter 1; LC3A, microtubule-associated protein 1 light chain 3α; LC3B, microtubule-associated protein 1 light chain 3β; MCT4, monocarboxylate transporter 4; n/a, not applicable; TNBC, triple-negative breast cancer.

### Western blot analysis of metabolism-related proteins in tumor and stroma according to tumor phenotype

**Figure 3:**
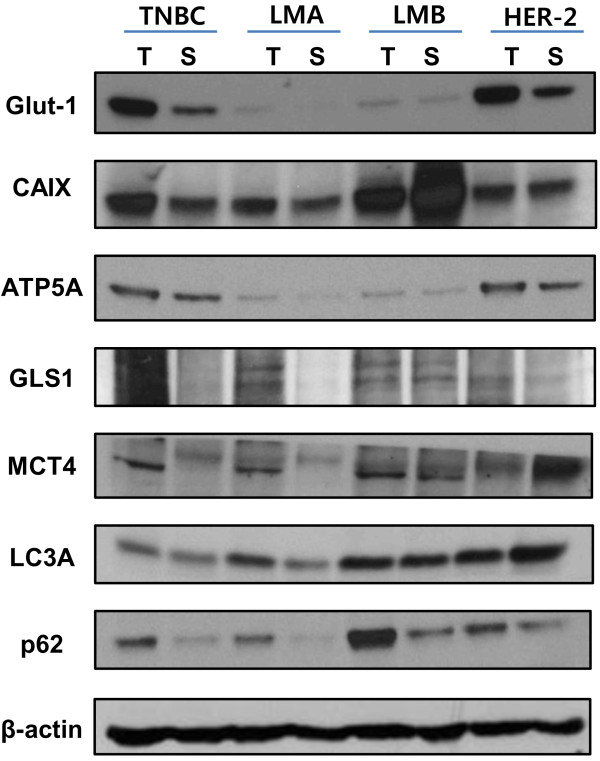
**Expression of metabolism-related proteins in tumor and stroma assessed by Western blot analysis according to tumor phenotype.** CAIX, carbonic anhydrase IX; GLS1, glutaminase 1; Glut-1, glucose transporter 1; LMA, luminal A; LMB, luminal B; LC3A, microtubule-associated protein 1 light chain 3α; MCT4, monocarboxylate transporter 4; S, stroma; T, tumor; TNBC, triple-negative breast cancer.

Western blot analysis was performed to investigate expression of metabolism-related proteins in tumor and stroma according to the tumor phenotype. The expression of Glut-1 and ATP synthase was higher in HER2 and TNBC types, and the expression was higher in tumor than in stroma (Figure 3). The expression of p62 was higher in tumor than stroma, regardless of the tumor phenotype.

## Discussion

In the present study, we investigated the differential expression of metabolism-related markers according to the subtypes of breast cancer. Expression of glycolysis markers such as Glut-1, CAIX and MCT-4 was highest in TNBC, which is consistent with the results of previous studies showing higher expression of Glut-1 and CAIX in basal-like breast cancer [19]. The active metabolic status of a tumor can be inferred from common histologic features of TNBC, such as high-grade nuclei, high-grade prominent necrosis and increased mitotic activity [20], and this was supported by the results of IHC in the present study. Tumor expression of Glut-1 was associated with higher histologic grade (*P* < 0.001), ER negativity (*P* < 0.001), higher T stage (*P* < 0.001), and higher Ki-67 LI (*P* < 0.001), whereas CAIX was associated with higher Ki-67 LI (*P* < 0.001). Tumor expression of MCT4 was associated with higher histologic grade (*P* < 0.001), ER negativity (*P* < 0.001), PR negativity (*P* < 0.001), higher T stage (*P* < 0.001) and higher Ki-67 LI (*P* < 0.001). In addition, expression of Glut-1, CAIX and MCT-4 was associated with factors reflecting higher metabolic status. In contrast, tumor expression of Glut-1, CAIX and MCT-4 was lowest in luminal A and B. Luminal type tumors tend to show a lower grade, lower mitotic index and less necrosis than the HER2 type or TNBC, suggesting nonactive metabolic status of the tumor, which was supported by the results of IHC.

The expression of Glut-1, CAIX, BNIP3, MCT4, LC3A, LC3B and p62 was observed not only in tumor cells but also in stromal cells, which has not been thoroughly described in previous studies. The reverse Warburg effect theory suggests that tumor stroma, along with the tumor *per se*, plays a role in cancer metabolism [2-5]. According to this theory, metabolism in stromal cells occurs through glycolysis due to dysfunctional mitochondria caused by increased autophagy, whereas metabolism of tumor cells occurs through OXPHOS in functional mitochondria. This contrasts with the conventional Warburg effect theory, which states that glycolysis is the major metabolic process in tumor cells. The major metabolic phenotypes in this study were the Warburg type (40.3%) and the null type (44.0%), according to the metabolic processes of tumor and stromal cells. We found that each metabolic phenotype investigated had different characteristics. The mixed type had higher histologic grade, ER negativity, PR negativity and higher Ki-67 LI, in contrast to the null type, which had lower histologic grade, ER positivity, PR positivity and Ki-67 LI (*P* < 0.001). As both tumor cells and stromal cells are glycolytic in the mixed type and nonglycolytic in the null type, we speculate that the mixed type is a group of tumors with high metabolic activity and that the null type consists of tumors with lower metabolic activity. The results of the present study show that the mixed type had the highest percentage of activated autophagy, whereas the null type had the lowest percentage, thus supporting this hypothesis. In addition, different molecular subtypes of breast cancer were classified into different metabolic types. TNBC constituted the highest percentage of Warburg type and mixed type, whereas the luminal A type constituted the highest percentage of reverse Warburg type and null type (*P* < 0.001). Moreover, the Warburg type and the mixed type were classified into groups with a higher Ki-67 LI, whereas the reverse Warburg type and the null type were classified into groups with a lower Ki-67 LI (*P* < 0.001). This result is consistent with those of a former study on the reverse Warburg effect in which a luminal A breast cancer cell line, MCF-7, was used for *in vitro* study [6]. Thus, further *in vitro* studies should be carried out with various cell lines showing different molecular subtypes.

We identified the expression of a mitochondrial metabolism-related protein such as ATP synthase and glutaminase in the tumor and stroma in the present study. Notably, stromal expression of ATP synthase and glutaminase was high in the reverse Warburg type and mixed type and low in the Warburg type and null type (*P* < 0.001). We speculate that stroma showing glycolysis have high mitochondrial metabolic activity, as both the reverse Warburg and mixed types are subtypes of the glycolysis type of stroma by definition. Because it has also been reported that certain types of tumor generate ATP through glycolysis as well as through mitochondrial OXPHOS, the hypothesis that dual types of stromal metabolism via the glycolysis pathway and the mitochondrial pathway should be investigated further.

The present study shows that the Warburg type and mixed type consisted of metabolically active and biologically aggressive tumors, whereas the reverse Warburg type and null type consisted of metabolically inactive and biologically nonaggressive tumors. This finding suggests that glycolysis of tumors significantly affects their metabolic and biological characteristics. The association of Glut-1 with shorter DFS and OS in univariate analysis supports this hypothesis.

A potential limitation of this study is the use of TMA cores for analysis, which may not truly represent the whole tumor. Although it is a reasonable contention, given the well-known intrinsic heterogeneity of breast cancer, this limitation was overcome by using two 3-mm tissue cores because it was previously reported that TMA with two 0.6-mm cores were representative of standard full tissue sections in breast cancer [21].

Among the breast cancer subtypes, TNBC comprised 28% of the total cases in this study, which is higher than the previously reported 12% to 24%. This difference can be attributed to possible differences in ethnic incidence, as reported previously, and to the overestimation of the true incidence potentially by the use of TMA containing part of the tumor, as we defined TNBC as all negative for ER, PR and HER2, which is similar to known phenomena of the discordance of ER, PR and HER2 expression between samples from core biopsy and excision [22-24]. Last, erroneous results of ER, PR and HER2 expression may affect the incidence, given that a 10% of false-negative rate and a 5% of false-positive rate were reported in ER expression, whereas a 4% of false-negative and false-positive cases were reported in HER2 [25]. Thus, cautious interpretation of the expression of those markers seems crucial, as misinterpretation of results may lead to the misclassification of the molecular subtypes.

## Conclusion

Breast cancer is heterogeneous in its metabolic status, and therefore it can be classified into various metabolic phenotypes. Specifically, the Warburg and mixed types had strong associations with TNBC, whereas the reverse Warburg type and the null type were associated with the luminal type, suggesting a correlation between metabolic phenotype and the biology of breast cancer.

## Abbreviations

ASCO: American Society of Clinical Oncology; CAF: Cancer-associated fibroblast; CAP: College of American Pathologists; FISH: Fluorescence *in situ* hybridization; H&E: Hematoxylin and eosin; LI: Labeling indices; NO: Nitric oxide; OXPHOS: Oxidative phosphorylation; ROS: Reactive oxygen species; TMA: Tissue microarray; TNBC: Triple-negative breast cancer.

## Competing interests

The authors declare that they have no competing interests.

## Authors’ contributions

JC participated in the design of the study, performed the statistical analysis and drafted the manuscript. DHK carried out the immunoassays and Western blot analysis. WHJ participated in the study design. JSK conceived the study, participated in its design and coordination and helped to draft the manuscript. All authors read and approved the final manuscript.
